# Efficacy of Percutaneous Laser Disc Decompression (PLDD) Combined with an Oral Food Supplement for Lumbar Disc Herniation

**DOI:** 10.3390/jcm13175049

**Published:** 2024-08-26

**Authors:** Roberto Gazzeri, Matteo Luigi Giuseppe Leoni, Felice Occhigrossi

**Affiliations:** 1 Interventional and Surgical Pain Management Unit, San Giovanni–Addolorata Hospital, 00184 Rome, Italy; focchigrossi@hsangiovanni.roma.it; 2 Department of Medical and Surgical Sciences and Translational Medicine, Sapienza University of Rome, 00100 Rome, Italy; matteolg.leoni@gmail.com

**Keywords:** percutaneous laser disc decompression (PLDD), neuropathic pain, herniated disc, lumbar disc, sciatica, lumbar disc herniation, oral food supplement, low back pain, dietary integrator, acetyl-L-carnitine, α-lipoic acid, quercetin, bromelain, pantothenic acid, B vitamins, MicronilDol

## Abstract

**Background**: In recent years, minimally invasive treatment options for lumbar disc herniation, such as percutaneous laser disc decompression (PLDD), have been introduced to avoid more invasive surgical methods. Combining these minimally invasive approaches with nutraceuticals that are effective in neuroprotection and pain management may lead to better long-term outcomes. **Methods**: The present study evaluated the beneficial effects of a new oral food supplement composed of acetyl-L-carnitine, α-lipoic acid, quercetin, bromelain, pantothenic acid, and vitamins C, B1, B2, B6, and B12 in patients with neuropathic pain due to herniated lumbar discs treated with PLDD. Patients were divided into two groups of 26 patients each: group A underwent PLDD alone, while group B underwent PLDD followed by a dietary supplement for two months after surgery. Preoperative VAS scores for leg pain were recorded for both groups and no significant difference was observed (8.7 for Group A and 8.6 for Group B). **Results**: In Group A, the mean postoperative VAS score for leg pain at a 1-month follow-up was 2.5, which remained stable at 3 months. In Group B, the mean postoperative VAS score was 2.0 at 1-month and improved to 1.6 at the 3-month follow-up. According to self-reported leg pain assessments, 66.5% of the patients using the dietary supplement reported a significantly better pain condition, and 43.5% reported a somewhat better situation. In contrast, 7.7% of the patients who underwent PLDD alone reported no changes in leg pain at the final follow-up. **Conclusions**: The results of our study indicate that the oral food supplement could provide a safe and effective treatment in patients with painful radiculopathy, enhancing the recovery of sensory fiber function in lumbar nerve roots after surgical lumbar disc decompression.

## 1. Introduction

Chronic low back pain (LBP) and sciatica caused by intervertebral disc herniation affect most adults and are the most common cause of disability and absence from work in the Western world. They impose significant personal, social, and economic burdens with a massive socioeconomic impact [1]. LBP is a common reason for seeking healthcare, being the second most frequent reason to visit a physician for a chronic condition and the third most frequent reason for undergoing a surgical procedure [2]. 

The approach to lumbar disc herniation is multidisciplinary, ranging from medical therapy and conservative treatment with physiotherapy to interventional approaches such as intradiscal techniques or herniated disc removal. In patients with neuropathic pain secondary to a lumbar herniated disc who remain symptomatic after conservative therapy, the standard approach of surgical decompression remains. Both open and percutaneous techniques are equally effective in patients with herniated discs [3].

In recent years, to avoid invasive surgical methods, percutaneous intradiscal procedures have been introduced [4]. With the aging of the population, combining minimally invasive approaches with nutraceuticals that are effective in neuroprotection and pain management may yield better outcomes over prolonged periods. Percutaneous laser disc decompression (PLDD) is a minimally invasive treatment option for lumbar disc herniation [5]. It is performed under local anesthesia using a laser fiber that is inserted percutaneously into the nucleus of the disc. The laser energy applied through the fiber causes the evaporation of water content inside the nucleus pulposus, resulting in the shrinkage of the disc due to water loss. This reduction in disc size and intradiscal pressure can lead to a reduction in nerve pressure. Proper patient selection is one of the most critical factors affecting the success rate of PLDD. 

The aim of this study was to evaluate the effectiveness of a new oral food supplement composed of acetyl-L-carnitine, α-lipoic acid, quercetin, bromelain, pantothenic acid, and vitamins C, B1, B2, B6, and B12 (MicronilDol^®^) in patients with neuropathic pain due to a herniated lumbar disc after PLDD. 

## 2. Materials

This study was a retrospective single-center clinical evaluation of patients older than 18 years with a herniated lumbar disc who underwent elective PLDD. All procedures were performed in accordance with the ethical standards of the institutional research committee and the 1964 Helsinki Declaration. Informed consent was obtained from all individual participants included in the study. Hospital records were reviewed, and patients’ demographic data, surgical indication, treatment levels, operative details, and complications were collected using standardized data collection forms. Patients were referred to us after failing extensive conservative treatment (physical therapy, anti-inflammatory medications, analgesics, and brace) for longer than 18 weeks. The minimum follow-up period for inclusion in this study was 10 months. Patients with a previous history of lumbar surgical interventions, severe stenosis, spondylolisthesis, spinal tumors, or infections were excluded from this study. 

Baseline data included patients’ age, sex, body mass index (BMI), smoking status, use of analgesics, and duration of symptoms. Pain was assessed using a visual analog scale (VAS) for leg pain, a quantitative rating scale where patients rate their pain from 0 (no pain) to 10 (maximum pain). Secondary outcomes included patient satisfaction, walking ability, and self-reported leg pain. Additionally, perioperative data and complications were recorded.

**Figure 1:**
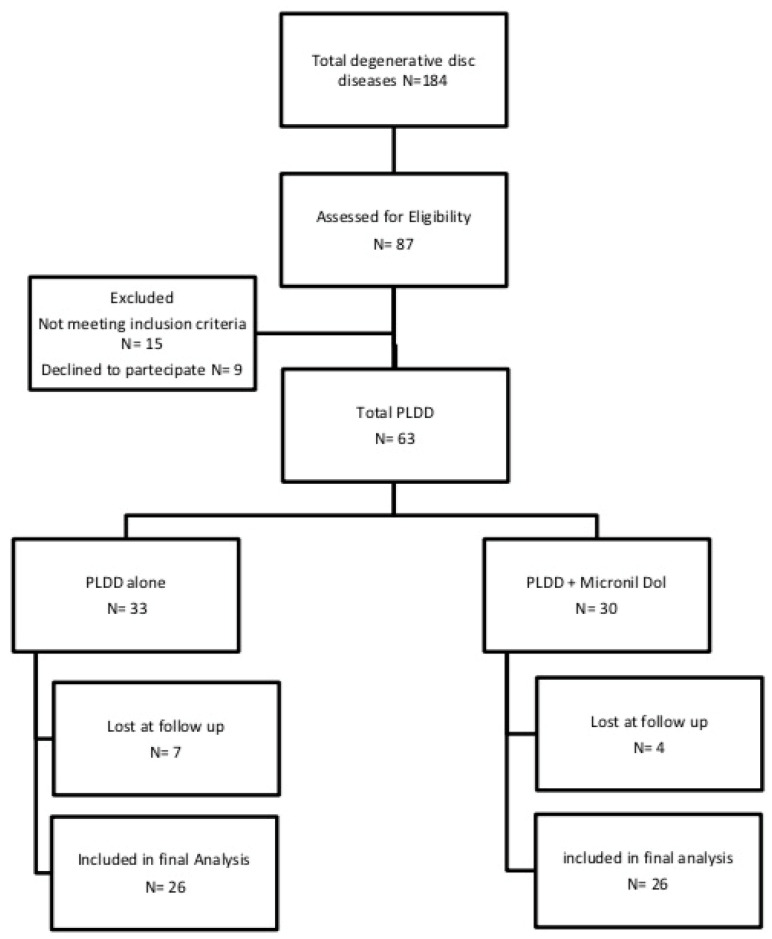
Flowchart of the study process. Twenty-six patients were included in each group of treatment at the final follow-up.

Patients were divided into two groups: group A (26 patients) underwent PLDD, and group B (26 patients) underwent PLDD followed by dietary supplement composed of acetyl-L-carnitine, α-lipoic acid, quercetin, bromelain, pantothenic acid, and vitamins C, B1, B2, B6 and B12 (MicronilDol^®^,Geopharma, Italy, 1 packet for 60 days). All patients were advised to avoid taking NSAIDs after surgery and were given the option of 1000 mg of paracetamol for the first postoperative week in case of excessive pain. (Figure 1). Clinical outcome assessments were performed three times: baseline (T0), 1 month after treatment (T1), and 3 months after treatment (T3).

## 3. Surgical Technique

With the patient placed in a prone position on a radiolucent table, a local anesthetic was administered, allowing the patient to cooperate. The entry point and trajectory of the needle were identified using an oblique fluoroscopic view, and the needle’s path was verified under fluoroscopic guidance. The final position of the needle tip within the center of the nucleus was confirmed by antero-posterior and lateral X-ray views. Intraoperative discography was performed to assess disc morphology alterations. A 400-micron optical fiber was inserted inside the needle, extending 1.00 mm beyond the needle tip. The optical fiber was then connected to a 1470 nm laser diode [NeoV (Neolaser, Caesarea, Israel)] previously calibrated to emit 7 watts at a 1.0 s burst (7 joules). Laser energy was delivered at 7-joule intervals with pauses of 1 to 3 s until reaching 800 joules. After irrigating the intradiscal space with 1 mg of gentamycin, the needle and fiber were removed. No sutures were used, and a band-aid dressing was applied.

## 4. Statistical Analysis

All the findings were entered into an Excel database (Microsoft Corp., Redmond, WA, USA) to ensure a consistent analysis of all parameters and to facilitate comparisons. A descriptive analysis of the clinical and demographic characteristics of patients was performed, reporting the mean and standard deviation. The association between variables was tested by unpaired *t*-test. A *p*-value < 0.05 was considered statistically significant. Statistical evaluations were performed using Excel software (Microsoft Corp., Redmond, WA, USA).

## 5. Results

### 5.1. Patients’ Population

In the present study, we included all patients who underwent PLDD for single- or double-level lumbar disc herniation during a two-year period (from January 2021 to January 2023) at the Interventional and Surgical Pain Management Unit of Azienda Ospedaliera San Giovanni Addolorata in Rome. Baseline data and a minimum of 10 months of follow-up were available for 52 patients. All baseline data were comparable in terms of demographic features and diagnoses (Table 1). The median age of the included patients was 62.3 ± 8.1 years in Group A and 64.1 ± 9.0 years in Group B, *p* = 0.37. They were mainly males, 54% and 58%, respectively. The median BMI was 21.8 ± 1.4 kg/m^2^ (SD) in Group A and 21.9 ± 1.5 kg/m^2^) in Group B, *p* = 0.75. The most common single-level PLDD was L4-L5 (13 Group A and 15 Group B), followed by L5-S1 single-level fixation (8 and 7 patients, respectively). A double-level PLDD was performed in three cases (5.8%). 

### 5.2. Preoperative Clinical Data

All included patients reported leg pain. Left-side radiculopathy was recorded in 10 patients (38.4%) in Group A and in 11 cases (42.3%) in Group B, *p* = 0.44, while right-side radiculopathy was observed in 11 and 10 patients respectively (*p* = 0.44). Five patients (19.2%) in each group reported bilateral radiculopathy. The duration of leg pain was 7.1 months in Group A and 7.9 months in Group B. Eighteen patients used analgesics regularly, while 21 used pain medications occasionally. Seven patients (32%) in Group A and eight patients (36%) in Group B were smokers (*p* = 1). There was no statistically significant difference between the two groups in preoperative VAS for leg pain (8.7 ± 1.2 in Group A and 8.6 ± 1.2 in Group B, *p* = 0.83). This lack of significant difference was also observed in the duration of leg pain (7.1 ± 3.2 months in Group A vs. 7.9 ± 3.4 months in Group B, *p* = 0.32) and preoperative walking ability between the two.

### 5.3. Postoperative Data

**Figure 2:**
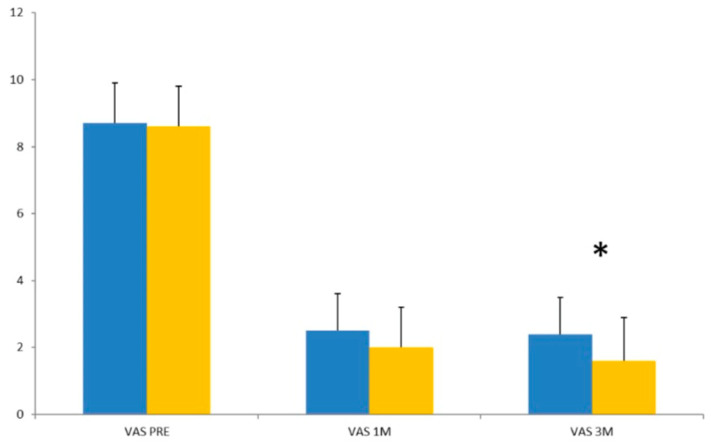
Preoperative (T0) and postoperative (T1 and T2) VAS score (* T TEST = *p* < 0.05). Blue = group A; yellow = group B.

**Figure 3:**
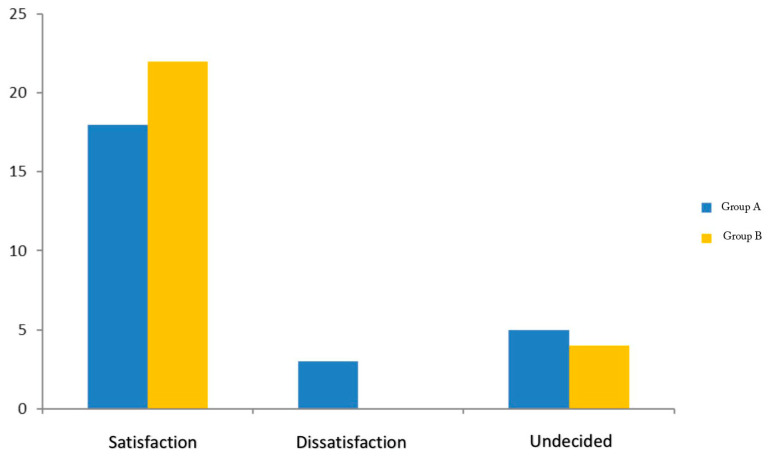
Patient satisfaction at final follow-up.

Operative time was comparable between the groups. However, although not statistically significant, Group A had a longer follow-up period compared to Group B (15 ± 4.4 months vs. 13.9 ± 3.9 months, *p* = 0.33). In Group A, the mean postoperative VAS leg pain score at the 1-month follow-up was 2.5 ± 1.1, which remained almost stable at the 3-month follow-up (VAS 2.4 ± 1.1). In Group B, the mean postoperative VAS score at 1 month was 2.0 ± 1.2, and at 3 months, it was 1.6 ± 1.3 (Figure 2). Self-reported leg pain showed a difference, with 66.5% of patients using dietary supplements reporting a much better pain condition and 43.5% a somewhat better situation, while 27% of the patients who underwent PLDD only reported a much better pain and 7.7% cases no change in leg pain. There were no operative and postoperative complications, such as nerve damage, dural injury, bleeding, or infection. A significant improvement in walking distance was observed in 31% (Group A) and 53.5% (Group B) of patients at the final follow-up. Patient satisfaction at the final follow-up showed 18 (69.2%) satisfied patients in group A and 22 (84.6%) patients in group B. Three patients (11.5%) in Group A were dissatisfied, while five patients (19.2%) in Group A and four patients (15.3%) in Group B were undecided (Figure 3).

## 6. Discussion

In this study, we sought to compare postoperative recovery in patients with neuropathic pain undergoing single or double-level PLDD combined with or without nutraceuticals. The analysis of our data showed a significant reduction in pain in patients who underwent PLDD. Furthermore, we found that a daily oral dose of 600 mg of a food supplement (acetyl-L-carnitine, α-lipoic acid, quercetin, bromelain, pantothenic acid, vitamins C, B1, B2, B6, and B12) for 2 months after surgery improved clinical outcomes following disc decompression.

Despite the development of various analgesic therapies and conservative approaches, managing LBP with radiculopathy remains a major concern. A major contributor to lumbosacral radiculopathy is neuropathic mechanisms caused by the compression and irritation of spinal root nerves from a lumbar disc herniation. One cause of nerve damage is increased oxidative stress, which may induce myelin degradation of nerve fibers. The inflammatory stress affects the lumbar nerves, leading to pain and loss of nerve functionality. Recently, percutaneous intradiscal procedures, which avoid open surgeries and hospitalization, were introduced for the treatment of disc herniation [6,7,8,9]. The intervertebral disc is a closed hydraulic system composed of over two-thirds water; vaporization of the nucleus with a laser slightly reduces the disc volume, leading to a significant decrease in intradiscal pressure [10]. Experimental studies have shown that a decrease in disc volume of only 1.0 mL reduces pressure by 312 kPa [11]. Laser application inside the intervertebral disc also leads to protein denaturation, limiting water resorption in the nucleus and reducing disc stiffness [12,13]. PLDD is a safe and effective surgical approach for selected cases of contained disc herniation with associated lumbar radiculopathy [14,15]. Although all patients with appropriate indications can benefit from this minimally invasive surgical approach, PLDD is also indicated for patients with comorbidities, obese cases, patients who cannot undergo general anesthesia, and those who do not want to undergo classic microdiscectomy. 

In the current study, we used a diode laser with a wavelength of 1460 nm. Regarding overall patient improvement, both the control group and the dietary integrated group showed significant improvements at 1 month and at 3 months compared to baseline. We found a significant decrease in VAS in both groups at 1 and 3 months after PLDD. In Group A, VAS decreased by 62% at 3 months follow-up, while a 70% reduction was observed in patients receiving a dietary integrator (MicronilDol^®^, Geopharma, Italy) postoperatively for 2 months. Although the difference between the two groups at the 1-month follow-up was not statistically significant (0.16), a significant difference was observed at the 3-month follow-up (*p* = 0.02). In our series, improvement in walking distance at three months follow up was significantly greater in the PLDD + nutraceutical group, increasing from less than 100 m to 500 m to more than 500 m to more than 1 km (53.5% vs. 31%). Preoperatively, in Group A, the walking distance was from less than 100 m to 500 m in 12 patients, while 14 patients could reach from 500 m to more than 1 km. After PLDD, walking distance in 22 cases was from 500 m to more than 1 km. Preoperative walking distance of patients receiving nutraceuticals was less than 100 m to 500 m in 16 cases, while 10 patients could reach a longer distance. At three months post-op, all except two cases in this group could walk from 500 m to more than 1 km. Patient satisfaction at follow-up showed higher satisfaction in patients who received oral food supplements (84% vs. 69.2%). A total of 11.5% of patients who did not receive integrators were dissatisfied, while 15.3% were undecided. 

Due to their analgesic effect, dietary integrators such as acetyl-L-carnitine, α-lipoic acid, quercetin, bromelain, and B complex vitamins have gained growing clinical interest for the treatment of various forms of chronic neuropathic pain. When the nerve root is repeatedly exposed to mechanical forces, demyelination of the nerve may occur. The mechanical effect of herniated disc material on neural tissue results in increased intraneural pressure, which may induce a decrease in blood supply to the endoneural capillary system, leading to alterations in the blood-nerve barrier and the development of intraneural edema [16,17,18]. The vicious circle of venous congestion, ischemia, hypoxia, and local metabolic alterations ultimately results in axonal degeneration, activation of macrophages, the release of inflammatory cytokines and nitric oxide, and, ultimately, tissue acidosis and chemical neuritis [19,20]. These pathophysiological alterations favor the degeneration of nerve cells, impair tissue regeneration, and compromise the recovery of neurological functions. Consequently, lesions in nervous tissue can cause neurological complications, such as neuropathic pain and sensory and motor deficits, with partial or total functional disability. This vicious circle may be interrupted by the simultaneous action of quercetin to counteract impaired microcirculation and vitamin B complex/α-lipoic acid/carnitine to improve nerve tropism. The neuroprotective action of these dietary integrators is exerted through several mechanisms. Several studies have provided evidence suggesting that alpha-lipoic acid (ALA) and other antioxidants exhibit neuroprotective effects in an experimental model of nerve compression [21,22]. In the nervous system, ALA is reported to have neuroprotective and antioxidant effects in various experimental and clinical studies [23]. 

Anti-inflammatory and antithrombotic mechanisms of α-lipoic acid improve endothelial function and blood flow to the nerves; moreover, ALA can regenerate endogenous antioxidants such as vitamins C and E, and regulate the transcription of genes associated with antioxidant and anti-inflammatory pathways [24,25]. Anxiety and depression are emotional dysfunctions associated with neuropathic pain; bromelain reduces pro-inflammatory mediators and has been reported to inhibit anxiety-like behaviors in the sciatic nerve ligation model of neuropathic pain [26,27]. Quercetin is a dietary flavonoid with antioxidant and anti-inflammatory potential, neuroprotective properties, and biological activity on nervous tissue [28,29,30]. Quercetin may have a beneficial effect on the nervous system, with the potential to minimize deleterious alterations, favor nerve tissue regeneration, and improve the recovery of neurological functions. Quercetin also modulated the inflammatory response and significantly inhibited oxidative stress and cellular apoptosis, favoring nerve fiber remyelination and improving sensory and motor recovery [31]. Quercetin has anti-inflammatory and antioxidant neuroprotective, immunoprotective, antiviral, and antibacterial properties [28,30]. The mechanism of action of quercetin involves protecting nervous tissue against oxidative damage resulting from physiological metabolism [32]. Furthermore, quercetin acts as an antioxidant, reducing ROS formation and lipid peroxidation and modulating the inflammatory response by inhibiting the synthesis of pro-inflammatory cytokines and favoring the synthesis of anti-inflammatory cytokines such as interleukin-10 [33]. In sciatic nerve injury models, quercetin administration inhibited the synthesis of pro-inflammatory cytokines, oxidative stress, and cell apoptosis, thus minimizing histopathological tissue damage and muscle atrophy [34]. Similarly, quercetin favored the synthesis of anti-inflammatory cytokines and the expression of growth-related genes, promoting axonal remyelination and neuronal regeneration, accelerating sensory and motor recovery, and improving locomotor capacity [34]. Acetyl-L-carnitine (ALC) provides a significant antinociceptive effect after the development of neuropathic pain. ALC has shown a neuroprotective effect in patients with peripheral neuropathies through several mechanisms. These analgesic properties result from different mechanisms, such as promoting the regeneration of injured nerve fibers, promoting DNA synthesis in mitochondria, reducing oxidative stress, and increasing NGF concentrations in neurons [35,36]. Neurotropic B vitamins may avoid the manifestation of peripheral neuropathy by directing the process of Wallerian degeneration to regeneration and remyelination. Vitamins B1, B6, and particularly B12 have nerve-regenerating effects individually, each via individual modes of action, but their combination creates the necessary environmental conditions for successful nerve regeneration, enabling synergies [37]. Vitamin B1 facilitates the energy production needed for the process and acts as a site-directed antioxidant, while vitamin B6 is vital for neurotransmitter synthesis and for inhibiting the release of neurotoxic glutamate [38,39]. Vitamin B12, on the other hand, largely promotes nerve cell survival and is strongly and directly involved in remyelination and the maintenance of myelin sheaths [40,41]. In general, the oral administration of nutraceuticals in humans is well-tolerated and safe. The novel multi-ingredient supplement used in this study allows for mixing a range of active ingredients in a single dosage sachet, improving patient compliance. 

There are some limitations in this study. The first is the small sample size, which does not allow the results to be generalized. The second limitation may be the choice of treating only contained herniated discs using the laser as the sole intradiscal technique, without evaluating other surgical approaches and stages of herniated discs. According to our results, the use of dietary integrators after surgical lumbar disc decompression may promote nerve function during the postoperative period. Further studies are mandatory with larger populations, and comparisons of different intradiscal techniques with various stages of disc herniation are mandatory.

## 7. Conclusions

The analysis of our data showed a significant reduction of pain in patients who underwent PLDD, with an even greater reduction in those receiving the dietary supplement (MicronilDol^®^) postoperatively for 2 months. These data indicate that oral supplements (composed of acetyl-L-carnitine, α-lipoic acid, quercetin, bromelain, pantothenic acid, and vitamins C, B1, B2, B6, and B12) could provide a safe and effective treatment for patients with painful radiculopathy, hastening the recovery of sensory fiber function in lumbar nerve roots after surgical disc decompression.

## Figures and Tables

**Table 1:** Patients’ characteristics.

	PLDD (Group A)	PLDD + Dietary Integrator (Group B)	Total
N° of cases	26	26	52
Gender (F/M)	12/14	11/15	23/29
Age	62.3 y.o.	64.1 y.o.	63.2 y.o.
BMI (kg/m^2^)	21.8	21.9	21.9
Smoker	7	8	15
Level Treated	L4-L5: 13 L5-S1: 8 L3-L4: 3 L2-L3: 1 L4-L5-S1: 1	L4-L5: 15 L5-S1: 7 L3-L4: 2 L4-L5-S1: 1 L3-L4-L5: 1	L4-L5: 28 L5-S1: 15 L3-L4: 5 L4-L5-S1: 2 L3-L4-L5: 1
Side	Right: 10 Left: 11 Bilateral: 5	Right: 11 Left: 10 Bilateral: 5	Right: 21 Left: 21 Bilateral: 10
Duration of leg pain (mths)	7.1	7.9	7.5
Analgesic Use	R: 15 S: 9 N: 2	R: 13 S: 12 N: 1	R: 28 S: 21 N: 3
Follow Up (mths)	15	13.9	14.4

## Data Availability

The original contributions presented in the study are included in the article, further inquiries can be directed to the corresponding author.
